# Robustness of Learning That Is Based on Covariance-Driven Synaptic Plasticity

**DOI:** 10.1371/journal.pcbi.1000007

**Published:** 2008-03-07

**Authors:** Yonatan Loewenstein

**Affiliations:** Departments of Neurobiology and Cognitive Sciences and Interdisciplinary Center for Neural Computation, Hebrew University, Jerusalem, Israel; University College London, United Kingdom

## Abstract

It is widely believed that learning is due, at least in part, to long-lasting modifications of the strengths of synapses in the brain. Theoretical studies have shown that a family of synaptic plasticity rules, in which synaptic changes are driven by covariance, is particularly useful for many forms of learning, including associative memory, gradient estimation, and operant conditioning. Covariance-based plasticity is inherently sensitive. Even a slight mistuning of the parameters of a covariance-based plasticity rule is likely to result in substantial changes in synaptic efficacies. Therefore, the biological relevance of covariance-based plasticity models is questionable. Here, we study the effects of mistuning parameters of the plasticity rule in a decision making model in which synaptic plasticity is driven by the covariance of reward and neural activity. An exact covariance plasticity rule yields Herrnstein's matching law. We show that although the effect of slight mistuning of the plasticity rule on the synaptic efficacies is large, the behavioral effect is small. Thus, matching behavior is robust to mistuning of the parameters of the covariance-based plasticity rule. Furthermore, the mistuned covariance rule results in undermatching, which is consistent with experimentally observed behavior. These results substantiate the hypothesis that approximate covariance-based synaptic plasticity underlies operant conditioning. However, we show that the mistuning of the mean subtraction makes behavior sensitive to the mistuning of the properties of the decision making network. Thus, there is a tradeoff between the robustness of matching behavior to changes in the plasticity rule and its robustness to changes in the properties of the decision making network.

## Author Summary

It is widely believed that learning is due, at least in part, to modifications of synapses in the brain. The ability of a synapse to change its strength is called “synaptic plasticity,” and the rules governing these changes are a subject of intense research. Theoretical studies have shown that a particular family of synaptic plasticity rules, known as covariance rules, could underlie many forms of learning. While it is possible that a biological synapse would be able to approximately implement such abstract rules, it seems unlikely that this implementation would be exact. Covariance rules are inherently sensitive, and even a slight inaccuracy in their implementation is likely to result in substantial changes in synaptic strengths. Thus, the biological relevance of these rules remains questionable. Here we study the consequences of the mistuning of a covariance plasticity rule in the context of operant conditioning. In a previous study, we showed that an approximate phenomenological law of behavior called “the matching law” naturally emerges if synapses change according to the covariance rule. Here we show that although the effect of slight mistuning of the covariance rule on synaptic strengths is substantial, it leads to only small deviations from the matching law. Furthermore, these deviations are observed experimentally. Thus, our results support the hypothesis that covariance synaptic plasticity underlies operant conditioning.

## Introduction

Synaptic plasticity that is driven by covariance is the basis of numerous models in computational neuroscience. It is the cornerstone of models of associative memory [1],[2],[3], is used in models of gradient estimation in reinforcement learning [4],[5],[6],[7],[8],[9],[10] and has been suggested to be the basis of operant conditioning [11]. In statistics, the covariance between two random variables is the mean value of their product, provided that one or both have a zero mean. Accordingly, covariance-based plasticity arises when synaptic changes are driven by the *product* of two stochastic variables, provided that the mean of one or both of these variables is subtracted such that they are measured relative to their mean value.

In order for a synapse to implement covariance-based plasticity, it must estimate and subtract the mean of a stochastic variable. In many neural systems, signals are subjected to high-pass filtering, in which the mean or “DC component” is attenuated relative to phasic signals [12],[13],[14],[15]. However, it is rare for the mean to be removed completely [16]. Therefore, while it is plausible that a biological synapse would be able to approximately subtract the mean, it seems unlikely that this mean subtraction will be complete. If mean subtraction is incomplete, the synapse is expected to potentiate constantly. Over time, this potentiation could accumulate and drive the synapse to saturation values that differ considerably from those predicted by the ideal covariance rule (see below). Thus, even if neurobiological systems actually implement approximate covariance-based plasticity, the relevance of the idealized covariance models to the actual behavior is not clear.

Here, we study the effect of incomplete mean subtraction in a model of operant conditioning, which is based on synaptic plasticity that is driven by the covariance of reward and neural activity. In operant conditioning, the outcome of a behavior changes the likelihood of the behavior to reoccur. The more a behavior is rewarded, the more it is likely to be repeated in the future. A quantitative description of this process of adaptation is obtained in experiments where a subject repeatedly chooses between two alternative options and is rewarded according to his choices. Choice preference is quantified using the ‘fractional choice’ *p_i_*, the number of trials in which alternative *i* was chosen divided by the total number of trials. The distribution of rewards delivered to the subject is quantified using the ‘fractional income’ *r_i_*, the accumulated rewards harvested from that alternative, divided by the accumulated rewards from all alternatives. In many such experiments, choice behavior can phenomenologically be described by
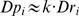
(1)where *i* = 1,2 corresponds to the two alternatives, *Dp_i_*≡*p_i_*−0.5 and *Dr_i_*≡*r_i_*−0.5. The proportionality constant, *k* corresponds to the susceptibility of choice behavior to the fractional income and its exact value has been a subject of intense debate over the last several decades. According to the ‘matching law’ *k* = 1 and thus *p_i_* = *r_i_*. In this case it can be shown that choices are allocated such that the average reward per choosing an alternative *i*, is equal for all alternatives [17],[18] (see also Materials and Methods). However, in many experiments the value of *k* is, in fact, slightly smaller than 1, a behavior that is commonly referred to as undermatching [19],[20],[21]. An alternative phenomenological description of behavior, known as ‘the generalized matching law’ [19] is *p*_1_/*p*_2_ = (*r*_1_/*r*_2_)*^k^*. Expanding the generalized matching law around *r_i_* = 0.5 yields Eq. (1) and thus Eq. (1) is an approximation of the generalized matching law. This approximation becomes equality for *k* = 1.

In a recent study we showed that the matching law is a natural consequence of synaptic plasticity that is driven by the covariance of reward and neural activity [11]. The goal of this paper is to understand the behavioral consequences of deviations from idealized covariance-based plasticity by investigating the behavioral consequences of incomplete subtraction of the mean in the plasticity rule. By studying an analytically solvable neural decision making model, we show that although the effect of small deviations from the idealized covariance-based plasticity on synaptic efficacies is large, the behavioral effect is small. Thus we demonstrate that matching behavior is robust to the mistuning of the parameters of the covariance-based plasticity rule. Furthermore, we show that the mistuning of the mean subtraction leads to undermatching, in line with experimental observations. Our study also reveals that the mistuning of the mean subtraction in the plasticity rule makes matching behavior sensitive to mistuning of the properties of the decision making network. Thus there is a tradeoff between robustness of matching behavior to changes in the plasticity rule and robustness to changes in the properties in the decision making network.

## Results

### The Decision-Making Model

**Figure 1:**
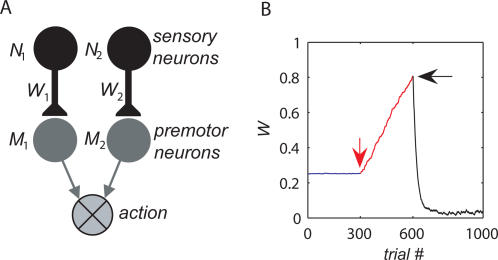
The model. (A) The decision making network consists of two populations of sensory neurons *N_i_*, corresponding to the two targets, and two populations of premotor neurons *M_i_*, corresponding to the two actions. Choice is determined by comparing the activities of the two populations of premotor neurons (see text). (B) The effect of the synaptic plasticity rule on synaptic efficacy. The decision making model was simulated in a concurrent VI reward schedule (see Materials and Methods) with equal baiting probabilities, and the efficacy of one of the synapses is plotted as a function of trial number. During the first 300 trials (blue), the synaptic efficacies evolved according to Eq. (2) with α = 0 and β = 1 (and thus γ = 0), resulting in small fluctuations of the efficacy around the initial conditions. A 10% mistuning of the mean subtraction after 300 trials (red arrow) to β = 0.9 (γ = 0.1) resulted in a linear divergence of the efficacy (red line). The addition of a linear decay term to the plasticity rule (Eq. (4) with ρ = 1) after 600 trials (black arrow) resulted in small fluctuations of the efficacy around 0.04 (black line).

Decision making is commonly studied in experiments in which a subject repeatedly chooses between two alternative actions, each corresponding to a sensory cue. For example, in many primate experiments, the stimuli are two visual targets, and the actions are saccadic eye movements to the targets [20],[21]. In our model, the responses to the sensory stimuli are represented by two populations of sensory neurons, whose level of activity is denoted by *N*_1_ and *N*_2_ (Fig. 1A). We assume that the two activities *N_i_* are independently drawn from the same Gaussian distribution with a positive mean and a coefficient of variation σ (standard deviation divided by the mean). We further assume that the level of variability in the activity of *N_i_* is low, σ≪1. This assumption is reasonable if *N_i_* corresponds to the average activity of a large population of uncorrelated neurons. Input from these sensory neurons determines the activities of two populations of premotor neurons via *M_i_* = *W_i_·N_i_* where *W_i_* corresponds to the synaptic efficacy of the sensory-to-premotor synapses. Competition between the two premotor populations determines whether the model will choose alternative 1 or 2 in a trial. Unless otherwise noted, alternative 1 is chosen in trials in which *M*_1_>*M*_2_. Otherwise alternative 2 is chosen. This process of competition between the two premotor populations can be achieved by a winner-take-all network with lateral inhibition [22], which is not explicitly modeled here. Thus, the larger the value of a synapse *W_i_* is, the more likely it is that alternative *i* will be chosen.

### Synaptic Plasticity

Consider the following plasticity rule, in which the change Δ*W_i_* in synaptic efficacy *W_i_* in a trial is described by
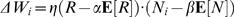
(2)where η is the plasticity rate, *R* is the reward harvested in the trial, **E**[*R*] is the average of the previously harvested reward, *N_i_* is the activity of sensory population *i* in the trial, and **E**[*N*] is the average activity of the sensory population. The index *i* is omitted from the latter average because we assume that the activity of the two populations is drawn from the same distribution; α, β are parameters. This plasticity rule corresponds to reward-modulated presynaptic activity-dependent plasticity [23],[24],[25]. If α = 1 and/or β = 1 then Eq. (2) describes a covariance-based synaptic plasticity rule because synaptic changes are driven by the product of two stochastic variables (*N_i_* and *R*) where the mean of one or both of these variables is subtracted. In order to gain insights into the behavior of Eq. (2), we consider the *average trajectory approximation*, also known as *mean synaptic dynamics* [26],[27],[28],[29], which is the dynamics of the expectation value of the right hand side of Eq. (2). If the plasticity rate η is sufficiently small, the noise accumulated over an appreciable number of trials is small relative to the mean change in the synaptic efficacies, called the synaptic drift [26],[27] and
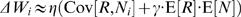
(3)where we define a mistuning parameter γ = (1−α)·(1−β). γ = 0 corresponds to the idealized covariance rule. Incomplete mean subtraction corresponds to γ>0. Our analysis focuses on choice behavior when mean subtraction is incomplete (γ>0). Similar results are obtained when mean subtraction is overcomplete (γ<0; see Materials and Methods). In principle, even a small mistuning of the mean subtraction may have a substantial effect on choice behavior for the following reason: Consider the dynamics of Eq. (3) for the simple case in which reward *R* and neural activity *N_i_* are independent. This corresponds to a case where the neural activity *N_i_* does not participate in the decision making process or to the case where reward is independent of choice. In both cases, Cov[*R, N_i_*] = 0 and therefore Eq. (3) becomes Δ*W_i_*≈η·γ·E[*R*]·E[*N*]. If E[*R*]·E[*N*]>0, the synaptic efficacy *W_i_* is expected to grow indefinitely. The divergence of the synaptic efficacies is also expected in the more general case in which the reward and neural activities are not independent. This is illustrated in Fig. 1B, where we simulated the plasticity rule of Eq. (2) in a concurrent variable-interval schedule (VI; see Materials and Methods) and plotted the efficacy of one of the synapses as a function of the trial number. When the covariance rule is finely tuned such that γ = 0 (here we assumed that α = 0, β = 1), the synaptic efficacy, after a transient period (not shown), is approximately constant (blue line). After 300 trials (red, down-facing arrow), the mean subtraction in the plasticity rule was mistuned by 10% such that γ = 0.9 (α = 0, β = 0.9), resulting in the linear divergence of the synaptic efficacy (red line).

In practice, synaptic efficacies are bounded and such divergence is prevented by synaptic saturation. We model the synaptic saturation by adding a polynomial decay term to the synaptic plasticity rule such that Eq. (2) becomes
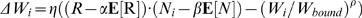
(4)where ρ>0 is the saturation stiffness parameter. The effect of the decay term on the dynamics of the synaptic efficacy is illustrated in Fig. 1B. After 600 trials (black, left-facing arrow), the plasticity rule of Eq. (2) was replaced with the plasticity rule in Eq. (4) with ρ = 1, resulting in a convergence of the synaptic efficacy to a value that is significantly different from the result of the pure covariance rule (black line).

The synaptic saturation is modeled here using a saturation stiffness parameter, ρ. When ρ = 1, as in Fig. 1B (black line), synaptic efficacies decay linearly. The larger the value of ρ, the stiffer the bound. In the limit of ρ→∞, as long as *W_i_*<*W_bound_* Eq. (4) is equivalent to Eq. (2), but the saturation term prevents *W_i_* from exceeding the value *W_bound_*.

### Incomplete Mean Subtraction

The dynamics of Eq. (4) are stochastic and therefore difficult to analyze. If the plasticity rate η is small then many trials with different realizations of choices and rewards are needed in order to make a substantial change in the value of the synaptic efficacies. Therefore intuitively, the stochastic dynamics of Eq. (4) can be viewed as an average deterministic trajectory, with stochastic fluctuations around it, where we expect that this average deterministic dynamics becomes a better approximation to the stochastic dynamics as the plasticity rate η becomes smaller. The conditions under which this intuitive picture is valid are discussed in [29]. The fixed point of the average trajectory of Eq. (4) is
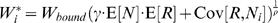
(5)and we study choice behavior when synaptic efficacies are given by Eq. (5). Assuming that *p*_1_, *p*_2_≠0, and γ>0, we show (Materials and Methods) that in the limit of low noise σ≪1, the model undermatches [19]; that is, when *p_i_*<0.5 then *p_i_*>*r_i_* whereas when *p_i_*>0.5 then *p_i_*<*r_i_*. Furthermore, the level of deviation from matching scales with the product of the mistuning and synaptic saturation parameters,
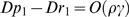
(6)Finally, expansion of Eq. (6) around *Dp_i_* = 0 yields Eq. (1) with
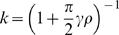
(7)Importantly, we show that overcomplete mean subtraction γ<0 also leads to undermatching with the same scaling of the deviations from matching with the mistuning and synaptic saturation parameters (Materials and Methods).

Consider Eq. (7). When γρ = 0, *k* = 1 and the fractional choice is equal to the fractional income yielding matching behavior. Note that when the mistuning of mean subtraction is small, γ≪1, the deviation of the susceptibility index *k* from 1 is small. This occurs despite the fact that such mistuning has, in general, a substantial effect on the values of the synaptic efficacies (Fig. 1B). Thus, matching behavior is robust to the mistuning of the mean subtraction, even though the synaptic efficacies are not.

#### The role of γ

For insights into the dependence of the susceptibility on γ, it is useful to consider the differential contributions of the covariance term, and the bias and saturation terms in Eq. (5). The smaller the value of γ, the larger the contribution of the covariance term, making it more similar to the idealized covariance-based plasticity rule that yields *k* = 1 [11]. In contrast, when the value of γ is large, the contribution of the covariance term is small and the efficacies of the two synapses, *W*_1_ and *W*_2_ become similar independently of the fractional income. In the limit of γ→∞, the efficacies of the two synapses become equal and the alternatives are chosen with equal probability. Thus, the larger the value of γ in Eq. (7), the smaller the susceptibility of behavior.

#### The role of ρ

Consider the case of an infinitely hard bound, ρ→∞ in Eq. (4). As long as *W* ^*^<*W_bound_*, (*W* ^*^/*W_bound_*)^ρ^ = 0. Because of the incomplete mean subtraction, the two synapses are expected to grow continuously until they reach *W_bound_*. For *W* ^*^>*W_bound_*, (*W* ^*^/*W_bound_*)^ρ^→∞. Thus both synaptic efficacies are expected to become equal to the synaptic bound *W_bound_*. In this case there is equal probability of choosing either alternative, independently of the fractional income, yielding *k* = 0. In contrast, a soft bound enables the saturation term to balance the bias term without occluding the covariance term. Thus, the smaller the value of ρ, the larger the contribution of the covariance term in the synaptic plasticity rule and the smaller the deviation from matching behavior.

#### The role of σ

In the limit of low noise in the activity of the sensory neurons σ≪1, choice behavior is independent of the value of σ. For insight into this independence we consider the dual role of trial-to-trial fluctuations in the neural activity of the sensory neurons in our model. Information about past incomes is stored in the synaptic efficacies such that the stronger synapse corresponds to the alternative that yielded a higher income in the past, biasing choice toward that alternative. For this reason we denote the difference in synaptic efficacies as ‘signal’. The trial-to-trial fluctuations in the neural activity of the sensory neurons underlie the stochasticity of choice. In the absence of such fluctuations, the synaptic efficacies determine choice such that the chosen alternative is the one that corresponds to the larger synaptic efficacy. The larger these fluctuations are the more random choice is. We refer to this effect as ‘noise’. However, these fluctuations also play a pivotal role in the learning process. Changes in synaptic efficacy are driven by the covariance of the reward and the neural activity of the sensory neurons. The larger the fluctuations in the activity of these neurons, the larger the covariance and therefore the larger the learning signal, increasing the difference between the synaptic efficacies that correspond to the “rich” and “poor” alternatives. Thus, an increase in the stochasticity in the activities of the sensory neurons increases both the signal and the noise. We show that when σ≪1, the ratio of the signal to noise is independent of σ (Materials and Methods) and therefore the susceptibility of behavior *k* is independent of σ.

### Numerical Simulations

**Figure 2:**
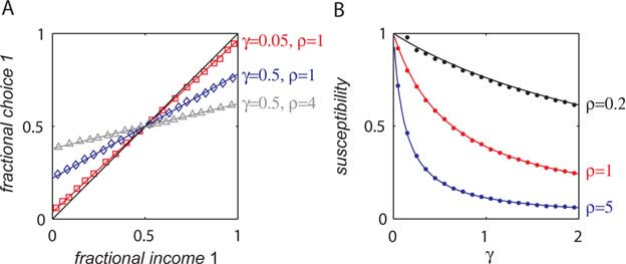
Incomplete mean subtraction and deviations from matching behavior. (A) The probability of choice as a function of fractional income. Each point corresponds to one simulation of the model, Eq. (4), in a concurrent VI reward schedule with fixed baiting probabilities. The level of deviation from matching behavior (black line) depends on the level of incomplete mean subtraction, γ and synaptic saturation stiffness, ρ. Red squares, γ = 0.05, ρ = 1; blue diamonds, γ = 0.5, ρ = 1; gray triangles γ = 0.5, ρ = 4; colored lines are the analytical approximations, Eq. (7). (B) Susceptibility of behavior as a function of γ. In order to quantify the effect of γ on deviation from matching behavior, we repeated the simulations of A for many values of γ and measured the susceptibility of behavior (the slope of the resultant curve, see text and Materials and Methods). Blue dots, ρ = 5; red dots, ρ = 1; black dots, ρ = 0.2. Lines correspond to the expected slope from the analytical approximation, Eq. (7).

Eq. (7) is derived assuming that the stochastic dynamics, Eq. (4) has converged to the fixed point of the average trajectory, Eq. (5) and that σ≪1 (Materials and Methods). In order to study the validity of this approximation, we numerically simulated the decision making model with σ = 0.1 and a stochastic synaptic plasticity rule, Eq. (4) in a concurrent VI reward schedule (Materials and Methods). These simulations are presented in Fig. 2. Each symbol in Fig. 2A corresponds to one simulation in which the baiting probabilities of the two targets were kept fixed. The fraction of trials in which action 1 was chosen is plotted against the fractional income earned from action 1. As predicted by Eq. (7), the dependence of the fractional choice on the fractional income is linear, and susceptibility depends on the values of both γ and ρ (red squares, γ = 0.05, ρ = 1; blue diamonds, γ = 0.5, ρ = 1; gray triangles γ = 0.5, ρ = 4; colored lines are the analytical approximation, Eq. (7); the black line is the expected behavior according to the matching law). In order to better quantify the relation between the stochastic dynamics and the analytical approximation, we simulated Eq. (4) for different values of γ and ρ and measured the susceptibility of behavior. The results of these simulations appear in Fig. 2B (blue dots, ρ = 5; red dots, ρ = 1; black dots, ρ = 0.2) and show good fit with the expected behavior from Eq. (7) (lines).

### Mistuning of Network Parameters

In the previous section we analyzed the behavioral consequences of mistuning of the plasticity rule in a particular network model. The question of robustness is equally applicable to the parameters of the decision making network as it is to the parameters of the synaptic plasticity rule. Therefore, in this section we study the robustness of matching behavior to the mistuning of the parameters of the network.

There are various ways in which the decision making network can be mistuned. We chose to study the effect of a bias in the winner-take-all network, because this is a generic form of error that is likely to significantly affect choice behavior. It is plausible that a winner-take-all network will be able to choose the alternative that corresponds to the larger activity of the two premotor populations in trials in which *M*_1_ and *M*_2_ are very different. However, if *M*_1_ and *M*_2_ are similar in their level of activity it is likely that a biological implementation of a winner-take-all mechanism, which is not finely tuned, will be biased to favoring one of the alternatives. Formally we assume that alternative 1 is chosen in trials in which (*M*_1_−*M*_2_)/(*M*_1_+*M*_2_)>ε where ε is a bias. The unbiased case studied in the previous section corresponds to ε = 0. In contrast, ε>1 or ε<–1 correspond to a strong bias such that choice is independent of the values of *M*_1_ and *M*_2_. With the same assumptions as in the derivation of Eq. (7), *p*_1_, *p*_2_≠0 and σ≪1, we show (Materials and Methods) that a bias in the winner-take-all mechanism results in a bias in choice that is *O*(ργ·ε/σ). Furthermore, analyzing choice behavior for small value of |*Dp_i_*| yields
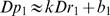
(8)where *k* is given by Eq. (7) and
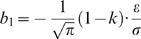
(9)is the offset. The offset *b*_1_ is proportional to the deviation of the susceptibility of behavior from unity, 1−*k*. As discussed in the previous section, this deviation depends on the level of incomplete mean subtraction as well as the synaptic saturation term (Eq. (7). If γ = 0 then *k* = 1 and the offset term vanishes, *b*_1_ = 0 for any value of bias ε. This robustness of matching behavior to bias in the winner-take-all network is due to the fact that the idealized covariance based plasticity rule can compensate for the bias in the decision making network in almost any neural architecture [11]. In contrast, if γ>0 then the offset *b*_1_ is proportional to the bias ε. The larger the deviation of the plasticity rule from the idealized covariance rule, the larger the proportionality constant. Thus, there is a tradeoff between the robustness of matching behavior to changes in the plasticity rule and robustness to changes in the parameters of decision making. The larger the mistuning of the plasticity rule, the smaller the robustness of matching behavior to mistuning of the parameters of the decision making network. Importantly, the level of noise in the sensory populations strongly affects the bias in behavior through ε/σ. This contrasts with the independence of the susceptibility parameter *k* of σ. To understand the reason for this result it is useful to note that as discussed in the previous section, the magnitude of trial to trial fluctuations in the activity of the sensory neurons determines the magnitude of the fractional income signal stored in the synaptic efficacies (the difference in the two synaptic efficacies). The smaller the value of σ is, the weaker the fractional income signal and therefore the stronger the relative contribution of the bias in the winner-take-all network to choice. If *N_i_* corresponds to the average activity of a large population of uncorrelated neurons, σ is expected to be small and therefore the effect of even small bias in the winner-take-all network on behavior is expected to be large.

### Numerical Simulations

**Figure 3:**
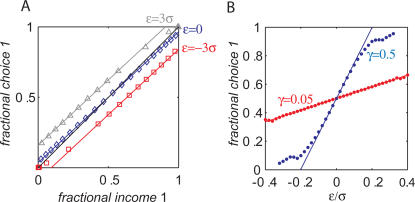
Bias in the winner-take-all mechanism and deviations from matching behavior. (A) The probability of choice as a function of fractional income. Each point corresponds to one simulation of the model (Eq. (4) with ρ = 1) in a concurrent VI reward schedule with fixed baiting probabilities. The level of deviation from matching behavior (black line) depends on the bias in the winner-take-all mechanism. Red squares, ε = −3σ; blue diamonds, ε = 0; gray triangle, ε = 3σ; γ = 0.05; colored lines are the analytical approximation, Eq. (8). (B) Choice bias. The simulation of A was repeated for different values of ε for two values of γ (blue dots, γ = 0.5; red dots, γ = 0.05), and the probability of choosing alternative 1 for a fractional income of *r*_1_ = 0.5 was measured. Lines correspond to the expected probability of choice from the analytical approximation, Eq. (8).

To study the validity of Eq. (8) numerically, we simulated the synaptic plasticity rule of Eq. (4) in the decision making model of Fig. 1A with a bias ε in the winner-take-all network. Similar to Fig. 2A, Fig. 3A depicts the fraction of trials in which alternative 1 was chosen, which is plotted against the fractional income earned from that alternative. The level of deviation from matching behavior (solid black line) depends on the value of ε (red squares, ε = −3σ; blue diamonds, ε = 0; gray triangle, ε = 3σ; γ = 0.05, ρ = 1). Colored lines are the analytical approximation, Eq. (8). In order to better quantify the relation between the stochastic dynamics and its deterministic approximation, we numerically computed the value of *p*_1_ that corresponds to δ*r*_1_ = 0 for different values of ε and γ (Fig. 3B; red, γ = 0.05; blue, γ = 0.5). The results are in line with the expected behavior from Eq. (8) (solid lines).

## Discussion

In this study we explored the robustness of matching behavior to inaccurate mean subtraction in a covariance-based plasticity rule. We have shown that (1) although this deviation from the idealized covariance rule has a substantial effect on the synaptic efficacies, its behavioral effect is small. (2) The direction of the behavioral effect of incomplete mean subtraction is towards the experimentally observed undermatching. (3) When the plasticity rule is mistuned, matching behavior becomes sensitive to the properties of the network architecture. Thus, there is a tradeoff between the robustness of matching behavior to changes in the plasticity rule and robustness to changes in the parameters of the decision making network.

### Robustness of Covariance-Based Plasticity

Covariance-based, Hebbian synaptic plasticity dominates models of associative memory. According to the popular Hopfield model, the change in the synaptic efficacy between pairs of neurons is proportional to the product of their activities in the training session, measured relative to their average activity [1],[2],[3]. If the mean subtraction is not finely tuned in this model, the synaptic efficacies diverge with the number of patterns stored. If this divergence is avoided by adding a saturation term to the plasticity rule, the capacity of the network to store a large number of memory patterns is lost [2],[30]. Thus, fine tuning of the mean subtraction in the plasticity rule is crucial for covariance-based associative memory models. This contrasts with the robustness of matching behavior to the mistuning of the mean subtraction demonstrated here. The difference in robustness stems from the difference in the solution space of the two tasks. Consider a general decision making network model consisting of *n* synapses. If *n*>1 the decision making model is expected to be redundant. There are many possible combinations of synaptic efficacies that yield the same probability of choice and thus are behaviorally indistinguishable. The dimension of the hyperspace of synaptic efficacies that corresponds to a single probability of choice is, in general, *n*−1. Consider now the hyperspace of synaptic efficacies that corresponds to the matching solution *p*_1_ = *r*_1_. Any set of synaptic efficacies that resides within this hyperspace is a fixed point of the family of synaptic plasticity rules that is driven by the covariance of reward and neural activity (in the average trajectory approximation) [11]. In contrast to this manifold of solutions, the approximate covariance plasticity rule with saturation is expected to have a single fixed point. In order for this fixed point to correspond to an approximate matching solution, it should reside near the matching hyperspace. The distance of the fixed point solution from the matching hyperspace depends on the decision making model and the level of mistuning of the covariance plasticity rule. However, because of the high dimensionality of the matching solution, there is a large family of decision making models in which the solution to the approximate covariance plasticity rule resides near the matching hyperspace for that model, for example, the model analyzed here with ε = 0. In contrast, in associative memory models, the volume in the synaptic efficacies hyperspace that can retrieve a large number of particular memories is small [31] and therefore even small deviations from the covariance plasticity rule will lead to a solution that is far from the memory retrieving hyperspace, resulting in a large reduction in the performance of the network.

Several studies have reported stochastic gradient learning in a model in which changes in the synaptic efficacy are driven by the product of the reward with a measure of past activity known as the ‘eligibility trace’ [4],[5],[6],[7],[8],[9],[10]. The mean of the eligibility trace is zero and therefore synaptic plasticity in these models can be said to be driven by the covariance of reward and a measure of past activity. Violation of the zero mean condition is expected to produce a bias in the gradient estimation and could potentially hinder learning. The consequences of mistuning of the mean subtraction in the estimation of the eligibility trace have not been addressed. We predict that the relative volume in the model parameter hyperspace that corresponds to the maximum reward solution will be an important factor in determining whether these gradient learning models are robust or not to the mistuning of the mean subtraction.

### Tradeoff between Sensitivity of Plasticity Rule and Network Architecture

The level of fine-tuning required for normal brain functioning is unknown and robustness represents a major open issue for many models of brain systems. For example, the fine-tuning of neural parameters involved in the short term memory of analog quantities such as eye position in the oculomotor neural integrator [32],[33],[34],[35] or the frequency of a somatosensory stimulation [36],[37] have been studied extensively. It has been suggested that synaptic plasticity keeps the synaptic efficacies finely-tuned [38],[39]. However, in those models it is assumed that the parameters of the plasticity rule are finely tuned. In this study we demonstrated a tradeoff between the robustness of behavior to changes in the parameters of the network architecture and the robustness to changes in the parameters of the plasticity rule. This tradeoff is likely to be a property of many models of brain function.

### Deviations from Matching Behavior

Undermatching in our model is the outcome of inaccurate mean subtraction, whether it is incomplete or overcomplete. This result is expected to hold in other symmetrical decision making models: when the mean subtraction is inaccurate, synaptic efficacies are determined by a combination of a covariance term, and bias and saturation terms. The bias and saturation terms are not influenced by the correlation between the neural activity and the reward. Therefore they drive the synaptic efficacies to values that are independent of the fractional income. If the architecture of the decision making network is symmetrical with respect to the two alternatives (as is the case in our model for ε = 0), they will drive the synaptic efficacies in the direction of a symmetrical solution for which the two alternatives are chosen with equal probability, which corresponds to *k*  = 0. In contrast, the covariance term drives the efficacies to the matching solution, *k* = 1. The combined effect of the covariance term and a small bias and saturation terms is expected to be a behavior for which the susceptibility index *k* is slightly smaller than 1, in line with the experimentally observed slight undermatching. Importantly, the experimentally observed undermatching is consistent with approximate covariance-based synaptic plasticity but does not prove it. Undermatching is also consistent with other models that do not assume this particular synaptic plasticity rule (see below).

### Experimental Predictions

We hypothesize that the observed matching behavior results from a synaptic plasticity rule that is driven by an approximation to the covariance of reward and neural activity. In this case, behavior adapts because synapses in the brain perform a statistical computation and ‘attempt’ to decorrelate the reward and the fluctuations in neural activity. However, a very different class of matching models has been proposed, in which the brain performs computations that are “financial.” According to these models, subjects keep track of financial quantities such as return or income from each alternative and make choices stochastically according to the difference or ratio of the financial quantities between the two alternatives leading to matching [20],[40],[41], or undermatching [42],[43]. A common feature of these models is the implicit assumption that financial computations and probabilistic choice are implemented in two separate brain modules. One brain module records past reward and choices to calculate quantities such as income and return and the other brain module utilizes these quantities to generate stochastic choice. A covariance-based plasticity rule can be distinguished experimentally from the financial models by making the reward directly contingent on fluctuations in the stochastic neural activity. This could be done by measuring neural activity in a brain area involved in decision making, using microelectrodes or brain imaging, and making reward contingent on these measurements, as well as on actions. This sort of contingency has previously been employed by neurophysiologists, though not in the context of operant matching [44],[45]. If, by the construction of the reward schedule, reward directly depends on fluctuations in neural activity, then it would be impossible to decorrelate the reward and the neural activity. According to our covariance hypothesis, the ‘attempt’ of the synaptic plasticity rule to do just this will lead to a change in the dependence of choice on the financial quantities (formally, this will lead to violation of Eq. (21) in Materials and Methods). In contrast, in the financial models, neural fluctuations and learning are mediated through different modules and therefore this contingency will not alter the dependence of choice on financial quantities (see also [11]).

## Materials and Methods

### Synaptic Efficacies and Choice Behavior

As was described above, the identity of choice in the network of Fig. 1 is determined by a competition between two premotor neurons *M_i_* = *W_i_·N_i_*. In the Incomplete mean subtraction section we assume that alternative 1 is chosen in trials in which *M*_1_>*M*_2_. Otherwise alternative 2 is chosen. Thus, the fraction of trials in which alternative 1 is chosen, or the probability that it is chosen is given by
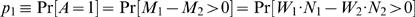
(10)where *A*∈{1,2} denotes the alternative chosen, or
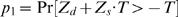
(11)where *Z_d_*≡(δ*N*_1_−δ*N*_2_)/(2·E[*N*]), *Z_s_*≡(δ*N*_1_+δ*N*_2_)/(2·E[*N*]), δ*N_i_* = *N_i_*−E[*N*], *T*≡*W_d_*/*W_s_*, *W_s_*≡(*W*_1_+*W*_2_)/2, *W_d_*≡(*W*_1_−*W*_2_)/2. Because *N*_1_ and *N*_2_ are independent Gaussian variables with a coefficient of variation σ, *Z_d_* and *Z_s_* are two independent Gaussian variables with zero mean and 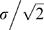 standard deviation. Therefore, *Z_d_*+*T*·*Z_s_* is a Gaussian variable with zero mean and 
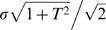
 standard deviation and
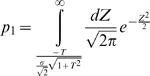
(12)Note that the assumption that *p*_1_,*p*_2_≠0 implies that in the limit of σ→0, *T* = *O*(σ).

Next we use Eq. (11) to compute two quantities that will become useful later:
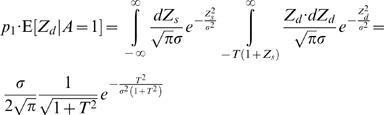
(13)and similarly
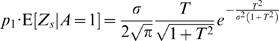
(14)Assuming that *T* = *O*(σ),
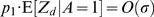
(15)and
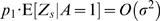
(16)


### Incomplete Mean Subtraction

In this section we compute the dependence of deviations from matching behavior on γ, assuming that synaptic efficacies are given by the fixed point of the average trajectory, Eq. (5). The precise conditions for the correctness of the approach are discussed in details in [29]. We further assume that synaptic saturation is linear, ρ = 1. The latter assumption is relaxed in the Incomplete mean subtraction and saturation stiffness section below.

According to Eq. (11), the probability of choice depends on the ratio of the synaptic efficacies; thus the scaling of the synaptic efficacies by a positive number does not change the probabilities of choice. For clarity we scale the synaptic efficacies of Eq. (5) (assuming ρ = 1) such that,
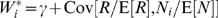
(17)Rewriting Eq. (17) in terms of *W_d_* and *W_s_* yields
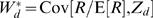
(18)

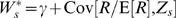
(19)where the asterisk corresponds to the value at the fixed point. Next we separate the covariance terms into trials in which alternative 1 was chosen and trials in which alternative 2 was chosen
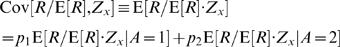
(20)The reward *R* is a function of the actions *A* and the actions are a function of the neural activities *Z_s_* and *Z_d_*. Therefore, given the action, the reward and the neural activities are statistically independent and the average of the product of reward and neural activity is equal to the product of the averages, E[*R*/E[*R*]·*Z_x_*|*A* = *i*] = E[*R*/E[*R*]|*A* = *i*]·E[*Z_x_*|*A* = *i*]. Hence, Eq. (20) becomes
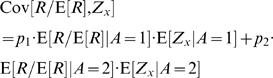
(21)Next we separate E[*Z_x_*] to trials in which alternative 1 was chosen and trials in which alternative 2 was chosen and use the fact that E[*Z_x_*] = 0
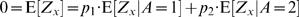
(22)Substituting Eq. (22) in Eq. (21) yields
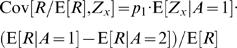
(23)In order to evaluate the second term in the right hand side of Eq. (23) we note that by definition, *r_i_* = *p_i_*·E[*R*|*A* = *i*]/E[*R*] and therefore,
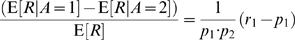
(24)where we assumed that *p*_1_,*p*_2_≠0 and used the fact that *p*_1_+*p*_2_ = 1 and *r*_1_+*r*_2_ = 1. Substituting Eqs. (13), (14), (23) and (24) in Eqs. (18) and (19) yields
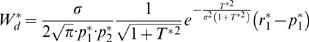
(25)and
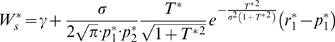
(26)where 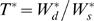. Combining Eqs. (25) and (26),
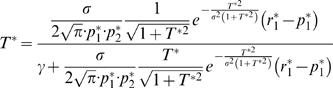
or
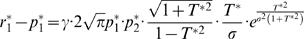
(27)Eq. (27) is central to this manuscript. Together with Eq. (12) which relates the probability of choice *p*_1_ with *T* it determines the level of deviations from matching behavior at the fixed point, 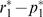 (The relation between *r*_1_ and *p*_1_ is determined by the reward schedule). Next we use Eq. (27) to show that:

1. In the limit of σ→0 the model undermatches.
2. The level of undermatching is proportional to γ, (Eq. (6)).
3. Expanding Eq. (27) around *p*_1_ = 0.5, yields a closed-form solution for *p*_1_ (Eq. (7)).

(1) As was discussed above, the assumption that *p*_1_,*p*_2_≠0 in the limit of σ→0 implies that *T* = *O*(σ)and therefore 1−*T* ^2^>0. Thus, 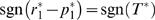. Using Eq. (12) and the notations of Eq. (1),
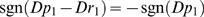
(28)(*Dp*_1_ and *Dr*_1_ in Eq. (28) are the values at the fixed point and therefore a more accurate notation would have included an asterisk. However, in order to keep notations in the text simple and notations in the Materials and Methods section consistent with the text we omitted the asterisk). When 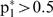, 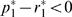 whereas when 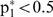, 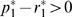. Thus we have shown that in the limit of σ→0 the model undermatches.

(2) Taking the dominant terms in σ in Eq. (27) yields
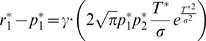
(29)
*T* ^*^ = *O*(σ) and thus the second term in the right hand side of Eq. (29) is *O*(1); therefore, the level of deviations from matching behavior is *O*(γ), Eq (6).

(3) In order to obtain a closed form approximation to Eq. (29) we expand Eq. (12) around *Dp*_1_ = 0 yielding
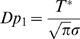
(30)Expanding Eq. (29) around *Dp_i_* = 0 and using Eq. (30) yields Eq. (7).

### Bias in Winner-Take-All Mechanism and Choice Behavior

In order to study the effect of bias in the winner-take-all network on choice behavior, we assume that that alternative 1 is chosen in trials in which (*M*_1_−*M*_2_)/(*M*_1_+*M*_2_)>ε where ε is a bias. Formally,
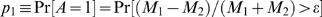
(31)Rewriting Eq. (31) in terms of *Z_s_* and *Z_d_* yields
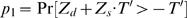
(32)where
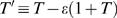
(33)or
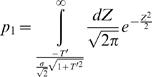
(34)The assumption that *p*_1_,*p*_2_≠0 implies in the limit of σ→0 *T*′ = *O*(σ). As in the derivation of Eqs. (13) and (14)
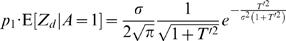
(35)and
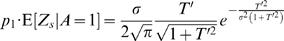
(36)Assuming that *T*′ = *O*(σ),
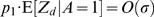
(37)and
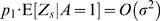
(38)From here we follow the same steps as in the derivation of Eq. (27) yielding
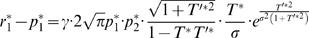
(39)or
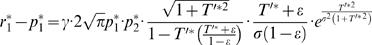
(40)Assuming that *T*′^*^ = *O*(σ) and taking the limit σ→0 yields
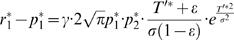
(41)Because *r*_1_−*p*_1_ = *O*(1), the assumption that 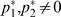 implies that γ·ε/σ = *O*(1). Thus in the limit of σ→0, ε≪1. Taking *O*(ε) terms in Eq. (41) yields
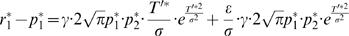
(42)The first term in the right hand side of Eq. (42) is equal to the right hand side of Eq. (29) and yields *O*(γ) deviations from matching behavior in the direction of undermatching. The bias in the decision making process, ε affects choice preference through the second term in the right hand side of Eq. (29). For *T*′ = *O*(σ), 
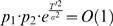
 and the contribution of the bias term ε to deviations from matching is *O*(γ·ε/σ).

Expanding Eqs. (34) and (42) around *Dp_i_* = 0 yields Eq. (8).

### Incomplete Mean Subtraction and Saturation Stiffness

Rewriting Eq. (5),
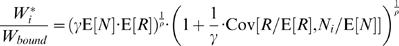
(43)Next we show that in the limit σ→0 and assuming that 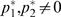, Cov[*R*/E[*R*],*N_i_*/E[*N*]]/γ≪1 and therefore the second term in the right hand side of Eq. (43) can be expanded around 1. In order to see this, we follow the same route as in the derivation of Eq. (23) and separate the covariance term into trials in which alternative 1 was chosen and trials in which alternative 2 was chosen
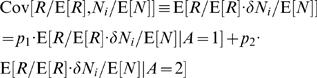
(44)As before, the reward *R* is a function to the actions, which in turn, are a function of the neural activity. Therefore, given the action *A*, *R* and δ*N_i_* are statistically independent and therefore
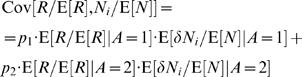
(45)By construction, E[δ*N_i_*/E[*N*]] = 0 and therefore,
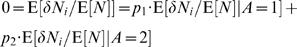
(46)Substituting Eq. (46) in Eq. (45) yields
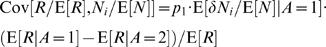
(47)Note that
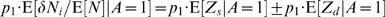
(48)Substituting Eqs. (16) and (15) in Eq. (48) yields,
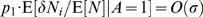
(49)Using Eq. (24), the assumption that 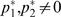 and taking the limit σ→0, such that σ/γ≪1 yields Cov[*R*/E[*R*],*N_i_*/E[*N*]]/γ≪1. In fact, substituting Eq. (6) in Eq. (24), Cov[*R*/E[*R*],*N_i_*/E[*N*]]/γ≪1 even when σ/γ↛0 as σ→0. Therefore, using self consistent arguments, the derivation of Eq. (50) is valid even when γ scales like σ. Expanding the second term in the right hand side of Eq. (43) yields,
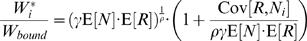
(50)According to Eq. (11), the probability of choice depends only on the ratio *W*_1_/*W*_2_. Therefore, the first term in the right hand side of Eq. (50) does not affect the probabilities of choice. The saturation stiffness parameter ρ affects the probability of choice through the second term and this effect is equivalent to the scaling of the mistuning parameter γ by ρ. Thus, assuming that synaptic efficacies converge to the fixed point of the average trajectory, Eq. (5), the effect of deviations of the saturation stiffness parameter from unity on choice is equivalent to the scaling of γ by ρ.

The synaptic saturation term also changes the effective plasticity rate, which will change the conditions of applicability of the average trajectory approximation. This analysis goes beyond the scope of this manuscript and will be discussed elsewhere. In short, changing the value of ρ changes the effective plasticity rate to 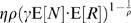. Therefore in the simulations in Fig. 2 we used
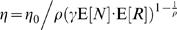
(51)


### Overcomplete Mean Subtraction and Saturation Stiffness

According to Eq. (3), when γ<0, *W_i_* is expected to depress until it becomes negative. In reality, synaptic efficacies are bounded and synaptic saturation prevents them from changing their sign. We model the synaptic saturation by replacing the synaptic plasticity rule of Eq. (2) by
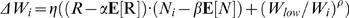
(52)where ρ>0 is the saturation stiffness parameter. The larger the value of ρ, the stiffer the bound. In the limit of ρ→∞, as long as *W_i_*>*W_low_* Eq. (52) is equivalent to Eq. (2), but *W_i_* is bounded from going below *W_low_*.

The fixed point of the average trajectory of Eq. (52) is
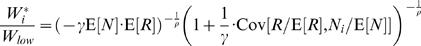
(53)Following the same steps as in the derivation of Eq. (50), the limit σ→0 with the assumption that 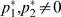 yields
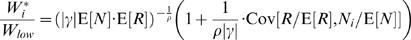
(54)Thus, assuming that synaptic efficacies converge to the fixed point of the average trajectory, Eq. (5), the behavior of a model with *overcomplete* mean subtraction is similar to that of a model with *incomplete* mean subtraction. In both cases the synaptic efficacies are given by
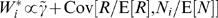
(55)where 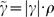


### Numerical Simulations

#### The reward schedule

The analytical results presented in this paper hold for a general diminishing-return reward schedule. They are demonstrated in the simulations using a concurrent VI reward schedule [19],[20]. On each trial, the subject chooses between two targets. If the chosen target is baited with reward, the subject receives it, and the target becomes empty. An empty target is rebaited probabilistically, according to the toss of a biased coin. Once baited, a target remains baited until it is chosen. Rewards are binary and no more than a single reward can reside in each target. Therefore, the reward schedule has two parameters: the biases of the two coins used to bait the targets. These biases, or baiting probabilities, control whether a target is “rich” or “poor.” A VI reward schedule has diminishing returns because a target is less likely to be baited if it has been chosen recently, as a consequence of the fact that reward persists at a target once the target is baited.

#### Simulation parameters

The sum of baiting probabilities in all simulations was kept constant at 0.5; σ = 0.1; E[*N*] = 1; plasticity rate in Fig. 1B is η = 0.05; plasticity rate in Figs. 2 and 3 is scaled according to Eq. (51) with η_0_ = 0.001. Each symbol in Figs. 2A and 3A corresponds to the average of 10^6^ trials of fixed baiting probabilities. Susceptibility was measured by computing the least-square-error linear fit.
